# Impact of Anesthetic Management on Safety and Outcomes Following Mechanical Thrombectomy for Ischemic Stroke in SWIFT PRIME Cohort

**DOI:** 10.3389/fneur.2018.00702

**Published:** 2018-08-29

**Authors:** Omer F. Eker, Jeffrey L. Saver, Mayank Goyal, Reza Jahan, Elad I. Levy, Raul G. Nogueira, Dileep R. Yavagal, Alain Bonafé, SWIFT PRIME investigators

**Affiliations:** ^1^Department of Neuroradiology, P. Wertheimer Hospital, Hospices Civils de Lyon, Lyon, France; ^2^Department of Neurology and Comprehensive Stroke Center, David Geffen School of Medicine, University of California, Los Angeles, Los Angeles, CA, United States; ^3^Department of Radiology and Clinical Neurosciences, University of Calgary, Calgary, AB, Canada; ^4^Division of Interventional Neuroradiology, University of California, Los Angeles, Los Angeles, CA, United States; ^5^Department of Neurosurgery, State University of New York at Buffalo, Buffalo, NY, United States; ^6^Marcus Stroke and Neuroscience Center, Grady Memorial Hospital, Department of Neurology, Emory University School of Medicine, Atlanta, GA, United States; ^7^Department of Neurology and Neurosurgery, University of Miami Miller School of Medicine–Jackson Memorial Hospital, Miami, FL, United States; ^8^Department of Neuroradiology, Hôpital Gui-de-Chauliac, CHU de Montpellier, Montpellier, France

**Keywords:** general anesthesia, conscious sedation, acute ischemic stroke, mechanical thrombectomy, blood pressure

## Abstract

**Background and purpose:** The optimal anesthetic management of acute ischemic stroke patients during mechanical thrombectomy (MT) remains controversial. In this *post-hoc* analysis, we investigated the impact of anesthesia type on clinical outcomes in patients included in SWIFT PRIME trial.

**Methods:** Ninety-seven patients treated with MT were included. Patients treated in centers with general anesthesia (GA) policy (*n* = 32) were compared with those treated in centers with conscious sedation (CS) policy (*n* = 65). Primary outcomes studied included times to treatment initiation (TTI), rates of successful recanalization (TICI 2b/3), and functional independence (mRS 0–2 at 90 days). Secondary outcomes were adverse events, lowest systolic and diastolic blood pressures (LSBP and LDBP) during MT. Univariate analysis and multivariate regression logistic modeling were conducted.

**Results:** The GA-policy and CS-policy groups presented comparable TTI (94 ± 36 min vs. 102 ± 48 min; *p* = 0.44), rates of TICI 2b/3 recanalization (22/32 [68.8%] vs. 51/65 [78.5%]; *p* = 0.32). CS-policy was associated to higher rate of functional independence than GA-policy, but the difference was not significant (43/65 [66.2%] vs. 16/32 [50.0%]; *p* = 0.18). GA-policy patients had a higher rate of postoperative pneumonia (11/32 [34.4%] vs. 8/65 [12.3%]; *p* = 0.02) and lower LSBP (110 [30,160] mmHg vs. 119 [77,170] mmHg; *p* = 0.03) and LDBP (55 (15,75) mmHg vs. 67 [40,121]; *p* < 0.001). When corrected for differences in baseline characteristics, GA-policy was associated with lower rate of functional independence (OR 0.32; *p* = 0.05). A 10-point increase in perprocedural LDBP was associated with an increased likelihood of favorable outcome (OR 1.51; *p* = 0.01).

**Conclusions:** GA-policy for MT presented comparable TTI and rates of successful revascularization to CS-policy. However, GA-policy was associated with lower rates of functional independence and with higher incidence of perprocedural hypotension and postoperative pneumonia.

**Clinical Trial Registration:** URL—http://www.clinicaltrials.gov. Unique identifier: NCT01657461

## Introduction

The benefits of endovascular mechanical thrombectomy (MT) for patients with anterior circulation acute ischemic stroke (AIS) in terms of reperfusion success, safety, and clinical outcomes have been demonstrated by randomized trials (1–5). However, the optimal anesthetic management of AIS patients during endovascular treatment remains unclear. Many authors supported general anesthesia (GA) as the preferred method (6, 7), whereas others advocated using conscious sedation (CS) (8–11). Most of these works were retrospective, heterogeneously designed and presented significant limitations, such as limited sample sizes, possible sampling bias, different and variable anesthetic drugs and/or blood pressure (BP) managements that make it difficult to draw definite and reliable conclusions (8–13). Recently, in three randomized clinical trial (RCT) on anesthetic management in patients with anterior circulation AIS treated by MT, showed no significant benefit of CS for MT compared to GA in terms of neurological status at 24 h, favorable clinical outcome at 90 days and infract growth and a significant tendency of GA to provide more functionally independent patients at 3 months, without any differences in mortality (14–16). The authors concluded that their findings did not support the posited advantages of using CS. In practice, anesthetic management is largely decided based on the local policies and preferences of the clinicians.

In this *post-hoc* analysis, we investigated the impact of anesthetic management during MT on clinical outcomes based on a review of the clinical data from the centers included in the SWIFT PRIME trial (2). The purpose of this study was to compare the rates of successful recanalization, procedural complications, and clinical outcomes including models appropriately corrected for differences in baseline characteristics, and the treatment initiation times (TTI) in anterior circulation AIS patients who received either GA or CS anesthetic management during MT.

## Materials and methods

### Study procedures

The present work is a *post-hoc* analysis of the Solitaire™ FR with the Intention for Thrombectomy as Primary Endovascular Treatment for Acute Ischemic Stroke (SWIFT PRIME) international, multicenter, prospective, and randomized clinical trial (2). The patients included in the endovascular treatment arm of the study (*n* = 98) treated with Solitaire Revascularization Device and IV-tPA were analyzed. The patients without any available anesthetic information were excluded. The impact of anesthetic management on the rates of successful recanalization, procedural complications, and clinical outcomes at 90 days post-procedure corrected for baseline characteristics, and workflow times were evaluated.

The primary analysis compared patients who were treated at hospitals where the policy was to perform MT under GA on all patients (GA-policy group) with patients treated at hospitals where the policy was to perform MT under CS on all patients whenever it was feasible (CS-policy group). Because of the possibility of non-standard anesthetic management with respect to the local policy (i.e., conversion to GA in CS-policy group and conversion to CS in GA-policy group), a secondary analysis compared all patients who actually received GA per the hospital routine or because of patient-specific requirements (i.e., neurological worsening, patient's agitation, need of airway protection; GA-delivered group) to those who received CS (CS-delivered group). A third analysis comparing the patients who received GA in GA-policy hospitals (GA by GA-policy) to those who received GA in CS-policy hospitals (GA by CS-policy because of patient patient-specific requirements) was conducted. A fourth analysis assessing interaction of anesthetic management with treatment effect was performed using data from the control group in SWIFT PRIME cohort (2).

The institutional review board at each site approved the trial. Either the enrolled patients provided written informed consent or, at select sites, there was an exception from the requirement for explicit informed consent due to emergency circumstances.

### Analyzed variables

The following clinical variables were collected: demographic data, blood glucose level, history of hypertension, dyslipidaemia, diabetes, cigarette smoking, and atrial fibrillation, severity of the neurological deficit according to National Institutes of Health Stroke scale (NIHSS) at admission, and clinical outcomes based on the modified Rankin Scale (mRS) score at the 90-day follow-up examination, which was assessed by a stroke neurologist not involved in the initial patient management. Imaging data were collected for the ischemic stroke side and intracranial occlusion site, including internal carotid artery (ICA) termination or middle cerebral artery (MCA, M1, or M2 segment). Baseline Alberta Stroke Program Early Computed Tomography (ASPECT) score was assessed either on computed tomography (CT) or on magnetic resonance diffusion-weighted imaging (DWI). Baseline infarct core volumes were assessed on either DWI or CT perfusion when performed and computed using RAPID software (iSchemaView, Menlo Park, CA) (17). Procedural data included the center's anesthetic policy (GA-policy vs. CS-policy), the type of delivered anesthesia (GA-delivered vs. CS-delivered), the rates of non-standard anesthetic management with respect to the local policy, lowest perprocedural systolic, and diastolic blood pressures (LSBP and LDBP), and the recanalization results according to Thrombolysis in Cerebral Infarction scale (TICI; successful recanalization defined as TICI 2b or 3) (18). Given the design of SWIFT PRIME trial, the used analgesics and sedatives and their respective doses, the reasons for non-standard anesthetic management (i.e., conversion), mean BP and the duration of any eventual hypotension during the MT were not recorded. The recorded workflow times included the time to treatment initiation (TTI) defined as the time from emergency room arrival (ER) to groin puncture, and the reperfusion time (RT) defined as the time from groin puncture to reperfusion. Procedural complications were recorded, including the presence of occlusive emboli in a different cerebral arterial territory, vessel dissection or perforation, intracranial hemorrhage (ICH), and device detachment. Adverse events including pneumonia, ICH during the first 24 h after randomization, and mortality at 90 days, were also recorded. ICH included subarachnoid hemorrhage (SAH), parenchymal hemorrhage (PH) categorized according to the European Cooperative Acute Stroke Study criteria (19). Symptomatic ICH (sICH) was defined as a CT- or MRI-documented hemorrhage associated with 4 or more points worsening on the NIHSS within 24 h after randomization.

### Statistical analysis

Continuous values are expressed as the median value and interquartile range or the mean ± standard deviation (SD) according to their distribution. Categorical variables are expressed as counts and percentages. Differences were analyzed using the Fisher's exact or Chi-square test for categorical variables, and using the Student *t*-test or Wilcoxon rank-sum test for continuous variables, according to their respective distributions. The TTI, rates of successful recanalization, and functional independence (mRS 0–2) at 90 days were compared as primary outcomes. Adverse events, rates of non-standard anesthetic management with respect to the local policy, perprocedural LSBP, and LDBP were compared as secondary outcomes. Multivariate models corrected for differences in baseline characteristics were created to examine multiple outcomes, including TICI 2b/3 and TICI 3 reperfusion scores, mRS 0–2 at 90 days, and the presence of SAH or PH. Each model produced corrected comparisons using the method of propensity scoring; propensity scores were defined using standard logistic regression methods against baseline characteristics including age, gender, history of diabetes mellitus, history of atrial fibrillation and hypertension, and NIHSS score at baseline. The effect of interest was included in each specific model (i.e., GA-policy vs. CS-policy and GA-delivered vs. CS-delivered). An mRS shift analysis was conducted comparing CS-policy and GA-policy groups. The mRS distribution in both groups were compared to that of the control group in SWIFT PRIME cohort (i.e., only IV tPA patients) (2). A logistic regression was employed to assess treatment effect on functional independence, separately for CS-policy centers and for GA-policy centers, and also to test for interaction of treatment group by GA policy vs. CS policy. A two-sided *p*-value < 0.05 was considered as statistically significant. Statistical analyses were performed using R version 3.2 (R Foundation for Statistical Computing, Vienna, Austria).

## Results

### Baseline data

Table 1 reports the baseline, procedural, outcome, and safety data for the GA-policy (*n* = 32, 33%) and CS-policy groups (*n* = 65, 67%). One patient never had groin puncture and has been excluded from the analyses. In GA-policy group, patients were significantly older (*p* = 0.03) and presented more often with hypertension (*p* = 0.02) than those in CS-group. Both groups presented comparable distributions in terms of the other baseline characteristics. Ten out of 97 patients (10.3%) were treated using non-standard anesthetic management with respect to the local policy. The rates of conversion to GA in CS-policy patients and to CS in GA-policy patients were comparable (7/65 [10.8%] vs. 3/32 [9.4%], respectively; *p* = 1). Thus, a total of 36 patients received GA among whom 7 (19.4%) were performed in CS-policy centers, and 61 received CS among whom 3/61 (4.9%) were performed in GA-policy centers. No patients were identified as receiving local anesthesia only.

**Table 1:** Baseline, procedural, safety, and outcome data for the CS-policy and GA-policy groups.

**Characteristics**	**CS-policy (*n* = 65)**	**GA-policy policy (*n* = 32)**	***p*-value**
Baseline			
Age, yrs.	63.1 ± 12.0 (65)	69.1 ± 12.7 (32)	0.03
Male sex	61.5% (40/65)	40.6% (13/32)	0.08
NIHSS score	16.8 ± 4.8 (65)	16.1 ± 4.0 (32)	0.44
ASPECTS score	8.5 ± 1.5 (65)	8.3 ± 1.5 (32)	0.52
Infarct core volume, mL	8.6 ± 11.5 (60)	14.2 ± 21.1 (26)	0.12
Risk factors			
Hypertension	60.0% (39/65)	84.4% (27/32)	0.02
Diabetes mellitus	13.8% (9/65)	9.4% (3/32)	0.75
Hyperlipidemia	24.6% (16/65)	25.0% (8/32)	1.00
Atrial fibrillation	29.2% (19/65)	50.0% (16/32)	0.07
Smoker	43.1% (28/65)	40.6% (13/32)	1.00
Glucose level, mg/dL	128.7 ± 46.7 (64)	135.5 ± 45.2 (32)	0.50
Occlusion site			
ICA termination	18.0% (11/61)	20.0% (6/30)	1.00
MCA	82.0% (50/61)	80.0% (24/30)	1.00
Procedural			
TTI, min	102 ± 48 (65)	94 ± 36 (32)	0.44
RT, min	43 ± 21 (65)	40 ± 13 (32)	0.61
LSBP, mmHg	119.0 (77.0, 170.0) (57)	110.0 (30.0, 160.0) (31)	0.03
LDBP, mmHg	67.0 (40.0, 121.0) (57)	55.0 (15.0, 75.0) (31)	0.001
Outcome			
mRS (ordinal)			0.66
0	18.5% (12/65)	15.6% (5/32)	
1	24.6% (16/65)	28.1% (9/32)	
2	23.1% (15/65)	6.2% (2/32)	
3	7.7% (5/65)	21.9% (7/32)	
4	13.8% (9/65)	18.8% (6/32)	
5	4.6% (3/65)	0.0% (0/32)	
6	7.7% (5/65)	9.4% (3/32)	
TICI 2b/3	78.5% (51/65)	68.8% (22/32)	0.32
TICI 3	60.0% (39/65)	56.2% (18/32)	0.83
NIHSS score at 27 h	8.2 ± 7.8 (65)	7.8 ± 5.9 (32)	0.80
Infarct core volume at 27 h, mL	59.2 ± 92.2 (64)	56.2 ± 66.4 (32)	0.87
mRS 0–2 at 90 days	66.2% (43/65)	50.0% (16/32)	0.18
mRS 6 at 90 days	7.7% (5/65)	9.4% (3/32)	1.00
Complications/Adverse events			
All ICH	24.6% (16/65)	31.3% (10/32)	0.63
sICH	0.0% (0/65)	0.0% (0/32)	1.00
SAH	1.5% (1/65)	0.0% (0/32)	1.00
PH2	4.6% (3/65)	15.6% (5/32)	0.11
Pneumonia	12.3% (8/65)	34.4% (11/32)	0.02
Vessel dissection	1.5% (1/65)	0.0% (0/32)	1.00

*ASPECT, Alberta stroke program early computed tomography score; CS, conscious sedation; GA, general anesthesia; ICA, internal carotid artery; ICH, intracranial hemorrhage; LDBP, lowest diastolic blood pressure; LSBP, lowest systolic blood pressure; MCA, middle cerebral artery; mRS, modified Rankin score; NIHSS, national institutes of health stroke scale; PH2, parenchymal hemorrhage grade 2 according to ECASS study (17); RT, reperfusion time; SAH, subarachnoid hemorrhage; sICH, symptomatic intracranial hemorrhage; TICI, thrombolysis in cerebral infarctions scale; TTI, time to treatment initiation; All values are shown as the mean ± SD (n), median (1st and third interquartile) or percentage (n/N)*.

### Procedural data

Both GA-policy and CS-policy groups presented similar TTI and RT. Compared with CS-policy group, GA-policy group presented significantly lower LSBP values (*p* = 0.03) and lower LDBP values (*p* = 0.001), as well as, greater reductions in pressure from baseline SBP (*p* = 0.05) and DBP (*p* = 0.02) during the procedure (Table 2). Vessel dissection occurred only in CS-policy group (*n* = 1, 1.5%) and no instances of vessel perforation, occlusive emboli, or device detachment were observed in any patients in either groups. The occurrence of any ICH did not significantly differ between both groups. No instances of sICH were observed. The rate of pneumonia was significantly higher in GA-policy group than in CS-policy group (*p* = 0.02).

**Table 2:** Perprocedural data in GA-policy and CS-policy groups after adjustment.

**Procedural data**	**Mean difference**	**95% Confidence interval**	***p*-value**
TTI, min	4.7	−36.3, 45.8	0.82
Perprocedural			
LSBP, mmHg	−15.2	−28.6, −1.9	0.03
LDBP, mmHg	−14.3	−22.6, −6.0	0.001
Change from baseline SBP, %	−12.8	−22.0, −0.3	0.05
Change from baseline DBP, %	−14.2	−25.8, −2.4	0.02

*LDBP, lowest diastolic blood pressure; LSBP, lowest systolic blood pressure; TTI, time to treatment initiation*.

### Clinical outcomes

**Figure 1:**
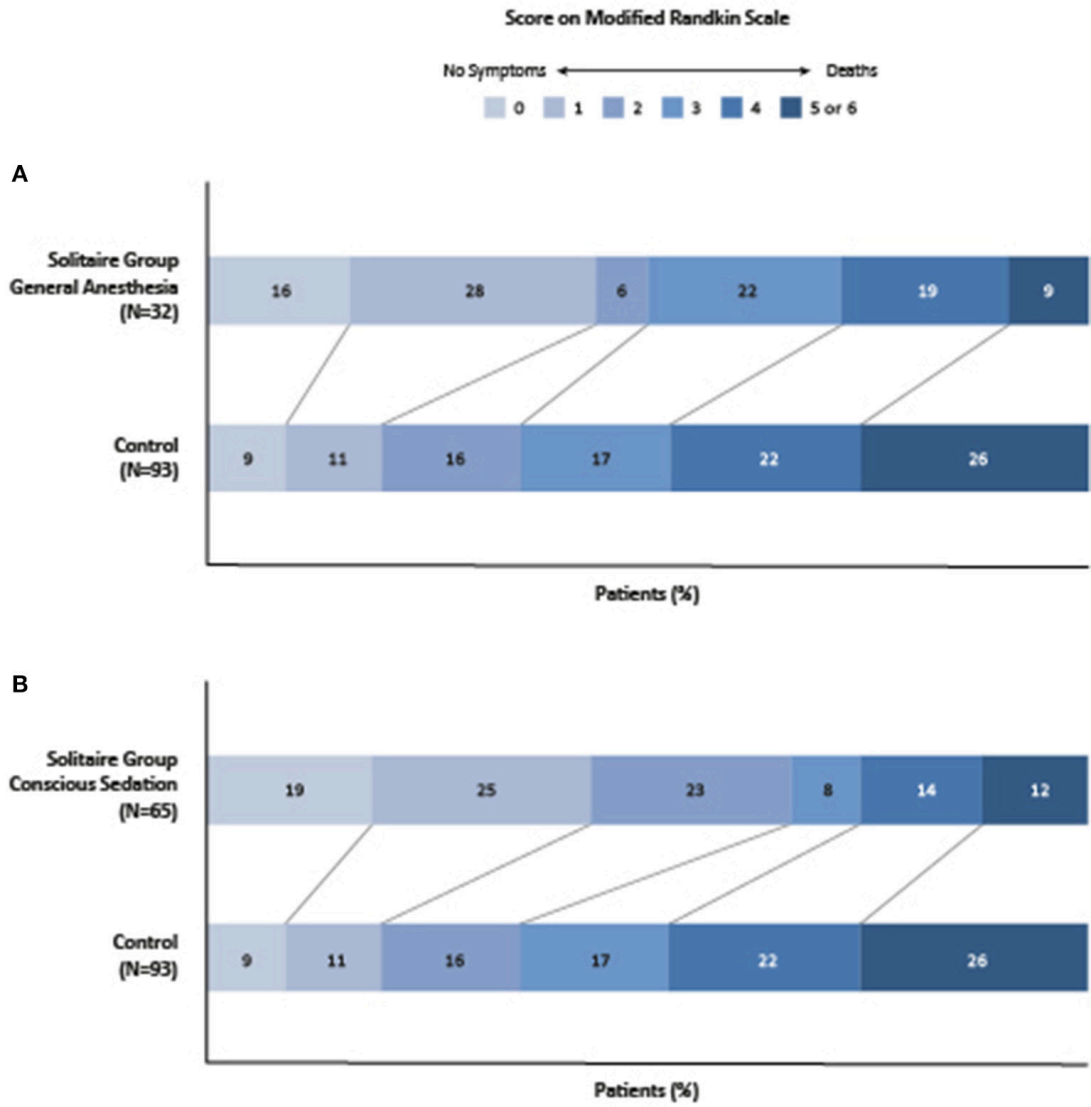
Functional outcomes at 90 days, according to the score on the modified ranking scale. The figure shows the subgroup analyses of mRS shifts comparison between GA-policy patients versus control group **(A)** and CS-policy patients versus control group **(B)** in SWIFT PRIME trial. Shown are the 90 day scores on the modified ranking scale for the patients in the two treatment groups. Score range from 2 to 6, with 0 indicating no symptoms, 1 no clinically significant disability (able to carry out all usual activities, despite some symptoms), 2 slight disability (able to look after own affairs without assistance but unable to carry out all previous activities), 3 moderate disability (requires some help but able to walk unassisted), 4 moderately several disability (unable to attend to bodily needs without assistance and unable to walk unassisted), 5 severe disability (requires constant nursing care and attention, bedridden, and incontinent), and 6 death.

GA-policy and CS-policy groups presented comparable rates of TICI 2b/3 recanalization (*p* = 0.32), similar distributions of NIHSS scores (*p* = 0.80) and infarct core volumes (*p* = 0.87) at 27 h. At 90 days, both groups presented comparable rates of mRS 6 (*p* = 1), and CS-policy group presented higher proportion of good functional outcome, although not statistically significant (*p* = 0.18). The mRS shift analysis compared to the control group is illustrated in Figure 1. It showed no significant differences in mRS distributions between CS-policy and GA-policy groups (*p* = 0.66). Compared to the control group, both the CS-policy group and the GA-policy group showed higher rates of functional independence (mRS 0–2): 66.2% [43/65] vs. 35.4% [23/65] (*p* = 0.001) for CS and 50.0 % [16/32] vs. 35.7% [10/28] (*p* = 0.31) for GA, without significant interaction between anesthetic management and treatment effect (*p* = 0.29).

In the logistic regression model, GA-policy was not a predictor of TICI 2b/3 recanalization, nor of any ICH or mortality at 90 days (Table 3). However, GA-policy was an independent predictor of pneumonia (OR 4.25; *p* = 0.03) and of lower rate of functional independence at 90 days (OR 0.32; *p* = 0.05). Independent from anesthetic management, an increase in the LDBP was an independent predictor of a mRS 0–2 at 90 days (OR 1.51 per 10 mmHg difference in LDBP; *p* = 0.01). Such a significant association was not seen between SBP and favorable clinical outcomes.

**Table 3:** Regression logistic model adjusted using the method of propensity scores comparing GA-policy with CS-policy as the independent variable of interest.

**Outcome**	Odds ratio^*^	**95% Confidence interval**	***p*-value**
Efficacy			
TICI 2b/3	0.64	0.13, 3.15	0.59
TICI 3	0.81	0.25, 2.61	0.72
mRS 0–2 at 90 days	0.32	0.10, 0.98	0.05
Safety			
SAH	0.85	NA	1.00
PH	2.23	0.36, 13.91	0.39
All ICH	1.39	0.44, 4.42	0.58
mRS 6 at 90 days	4.90	0.49, 26.77	0.11
Adverse events			
Pneumonia	4.25	1.14, 15.78	0.03

*ICH, intracranial hemorrhage; mRS, modified Rankin Scale; NA, not applicable; PH, parenchymal hematoma; SAH, subarachnoid hemorrhage; TICI, thrombolysis in Cerebral Infarction scale*.

* *The odds ratio reflects the odds of the General Anesthesia group in comparison with the Conscious Sedation group, so values <1 indicate a reduced likelihood of the event with General Anesthesia compared with Conscious Sedation group*.

The results of the analyses based on the actually delivered anesthetic management (i.e., GA-patients vs. CS-patients) are reported in the online-only supplemental data (Supplemental Tables 1–3). These analyses provide similar results for univariate and for logistic regression model to those based on the local anesthetic management policy in terms of baseline, procedural and outcome and safety data, apart from two points: (1) both groups presented comparable mean ages (67.4 ± 12.2 vs. 63.7 ± 12.6, respectively; *p* = 0.16); and (2) despite a higher rate of pneumonia in the GA-patients group, the difference did not reach any statistical significance (11/36 [30.6%] vs. 8/61 [13.1%], respectively; *p* = 0.06).

The results of the analyses comparing the GA delivered in GA-policy (*n* = 29) to GA delivered in CS-policy (*n* = 7) are reported in Table 4. These analyses showed no significant differences between both groups for baseline, procedural, safety, and outcome data, apart from, a significantly longer ER arrival to reperfusion time associated to GA given because of conversion from CS (*p* = 0.01). The GA by CS-policy group showed a higher proportion of good functional outcomes than the GA by GA-policy group, although it did not reach any statistical significance (15/29 [51.7%] vs. 6/7 [85.7%], respectively; *p* = 0.20).

**Table 4:** Baseline, procedural, safety and outcome data for the GA by GA-policy group and GA by CS-policy group.

**Characteristics**	**GA by GA-policy**	**GA by CS-policy**	***p*-value**
Baseline			
Age, yrs.	68.2 ± 12.9 (29)	64.1 ± 8.8 (7)	0.44
Male sex	41.4% (12/29)	71.4% (5/7)	0.22
NIHSS score	16.0 ± 4.2 (29)	18.4 ± 3.6 (7)	0.18
ASPECTS score	8.2 ± 1.5 (29)	9.0 ± 1.4 (7)	0.21
Risk factors			
Hypertension	86.2% (25/29)	85.7% (6/7)	1.00
Diabetes mellitus	6.9% (2/29)	0.0% (0/7)	1.00
Hyperlipidemia	24.1% (7/29)	28.6% (2/7)	1.00
Atrial fibrillation	48.3% (14/29)	28.6% (2/7)	0.43
Smoker	37.9% (11/29)	83.3% (5/6)	0.07
Glucose level, mg/dL	136.9 ± 46.9 (29)	136.8 ± 41.2 (6)	0.99
Occlusion site			0.40
ICA termination	14.8% (4/27)	33.3% (2/6)	
MCA	85.2% (23/27)	66.7% (4/6)	
Procedural			
Onset to qualifying image, min	163.6 ± 82.2 (29)	132.0 ± 67.3 (7)	0.35
ER arrival to reperfusion, min	134.7 ± 40.8 (21)	194.2 ± 68.5 (6)	0.01
LSBP, mmHg	109.0 (30.0, 150.0) (28)	118.0 (90.0, 124.0) (5)	0.53
LDBP, mmHg	55.0 (15.0, 75.0) (28)	72.0 (50.0, 77.0) (5)	0.12
Outcome			
TICI 2b/3	83.3% (20/24)	83.3% (5/6)	1.00
TICI 3	66.7% (16/24)	50.0% (3/6)	0.64
NIHSS score at 27 h	8.1 ± 6.0 (29)	11.9 ± 9.2 (7)	0.19
mRS 0–2 at 90 days	51.7% (15/29)	85.7% (6/7)	0.20
mRS 6 at 90 days	10.3% (3/29)	0.0% (0/7)	1.00
Complications/Adverse events			
All ICH	31.0% (9/29)	14.3% (1/7)	0.64
sICH	0.0% (0/29)	0.0% (0/7)	1.00
SAH	0.0% (0/29)	0.0% (0/7)	1.00
PH2	13.8% (4/29)	0.0% (0/7)	0.57
Pneumonia	34.5% (10/29)	14.3% (1/7)	0.40
Vessel dissection	0.0% (0/29)	0.0% (0/7)	1.00

*ASPECT, Alberta stroke program early computed tomography score; CS, conscious sedation; ER, emergency room; GA, general anesthesia; ICA, internal carotid artery; ICH, intracranial hemorrhage; LDBP, lowest diastolic blood pressure; LSBP, lowest systolic blood pressure; MCA, middle cerebral artery; mRS, modified Rankin score; NIHSS, national institutes of health stroke scale; PH2, parenchymal hemorrhage grade 2 according to ECASS study (17); SAH, subarachnoid hemorrhage; sICH, symptomatic intracranial hemorrhage; TICI, thrombolysis in cerebral infarctions scale; All values are shown as the mean ± SD (n), median (1st and third interquartile) or percentage (n/N)*.

## Discussion

Our study shows that GA and CS during MT for the treatment of AIS, either as local anesthetic policy or as actually delivered anesthetic management, had comparable TTI and rates of successful recanalization. Our results also suggest a higher proportion of pneumonia as adverse event and a lower proportion of favorable clinical outcomes at 90 days associated with GA (as policy or delivered management). Although the differences were not statistically significant, the adjusted regression analysis supported this suggestion as GA (as policy or delivered management) was a significant independent predictive factor of these two outcomes. Similarly, GA-policy was significantly associated with a lower perprocedural LDBP. The relative increases in perprocedural LDBP seen with CS (as policy or delivered management compared to GA) was significantly associated with favorable clinical outcomes at 90 days.

Our patient outcomes should be interpreted with the knowledge that compared to CS-policy patients, GA-policy patients had more often history of hypertension. Comparing GA to CS is subject to strong “confounding by indication” bias (i.e., sampling bias), which was one of the main limitations of previous works. In order to mitigate this, we differentiated the primary analysis based on local anesthetic policies from the second analysis based on the actually delivered anesthetic management. In addition, we conducted an analysis using favorable functional outcome at 90 days as the outcome, comparing treatment vs. control arms in SWIFT PRIME cohort and distinguishing the GA-policy and CS-policy patients. It showed a benefit of treatment in both groups, with a statistical significance only in CS-policy group. However, the test of interaction (i.e., treatment by policy) was non-significant. Therefore, we should assume similar treatment effect under GA, although the limited numbers in each subgroups prevent to draw any definite conclusions.

Our results agree in part with some previously published works, since pneumonia and low BP are known to promote poor outcomes (13, 20). Therefore, it is possible that the effect of GA on clinical outcomes may be partly due to the significantly lower BP during MT. However, our study further suggests that (1) only the LDBP seems to impact the clinical outcome, whereas the effect of the LSBP was not significant, and (2) a 10-point increase in LDBP was associated with a favorable outcome at 90 days (*p* = 0.01). This is in good agreement with the results from ANSTROKE trial and offers prospects for BP optimization under GA for MT by focusing on the increase in DBP (15).

Our *post-hoc* analysis allowed also a comparison analysis between GA-patients treated in GA-policy centers vs. those treated in CS-policy centers. Interestingly, both groups presented no significant differences in baseline, procedural, safety, and outcome data variables, apart from, a significantly longer ER arrival to reperfusion time associated to GA given in CS-policy centers. Our work contributes to the ongoing and controversial discussion in the literature concerning the anesthetic management of AIS patients during MT, in that it demonstrates the lack of the higher procedural complication rates for CS that was predicted by its opponents (11). It also support retrospective studies in support of CS which have asserted that its use avoids a drop-in BP and worse clinical outcomes (6, 7), despite recent RCTs that did not support these conclusions (14–16). Contrary to what was previously reported, GA-policy did not significantly delay the TTI when it was delivered in GA-policy centers (i.e., 9 min in the GA-policy group vs. 32 min in MR CLEAN, 10 min in SIESTA trials) (14, 21). This result is in good agreement to that of previously reported RCTs on anesthetic management. They suggested that the longer time between patients arrival and treatment initiation is compensated by the shorter time between groin puncture and reperfusion in GA. On contrary, we observed that GA delivered in CS-policy centers was associated to a delayed ER arrival to reperfusion time. This might be explained by the delay due to conversion from CS or because of patients requirements in centers not using GA as protocol for MT. This suggest that GA does not delay TTI when it is delivered in centers where it is delivered as routine anesthetic management.

In agreement with the results of the SIESTA and ANSTROKE trials, as well as the HERMES meta-analysis, we found a higher rate of pneumonia as an adverse event associated with GA management during MT (14, 15, 22). The SIESTA, ANSTROKE, and GOLIATH trial found no differences between the GA and CS groups in term of favorable outcomes (either as NIHSS, mRS at 90 days or infract growth, respectively), with a tendency for patients to demonstrate better outcomes with GA (14–16), which is the opposite of what we report. On contrary, our result support those of HERMES meta-analysis with regard to lower rates of favorable outcome associated with GA (22). For this discrepancy, four reasons related to the limitations of our study are possible. First, all the three trials randomized for anesthetic management in MT, both CS and GA arms likely had comparable and lower incidences of low BP than the GA groups (as policy or delivered management) in our study, because they used well-developed and aggressive policy to manage BP. Second, in all of the three randomized trial both CS and GA groups received highly protocol-specified approach with intravenous, short acting analgesics and sedatives. Especially in SIESTA trial, the first group received them at low-dose whereas the second group received them at higher doses. In SWIFT PRIME trial, the method of GA was at the discretion of the treating team without formal protocol specifying the drugs to use, the BP targets or other aspects of physiological management. In particular, any impairment of the collateral blood flow to the ischemic penumbra cannot be ruled out in our GA-patients because of the absence of protocolized strict attention to maintaining BP during the endovascular procedure. Third, confounding by indications (i.e., sampling bias) in our study is another potential contributor to our results, as in HERMES meta-analysis. We sought to mitigate this possibility by analysing GA-policy vs. CS-policy groups of patients. However, it is possible that our identification of patients in each of these categories was not fully accurate and may not have corrected for the bias. Fourth, our patients' population has been included in the HERMES meta-analysis, representing around 12% of the global analyzed population. However, our results differs from HERMES ones in that GA was not associated with delay in treatment initiation or reperfusion (22).

It is worth mentioning that our cohort presented a higher conversion rate from CS to GA compared with previously reported works (13, 23), with nearly 20% (7/36) of GA patients receiving a non-standard anesthetic management with respect to local policy (i.e., CS-policy). Unfortunately, the reasons for these conversions were not recorded, meaning that any deteriorations of the patients who required an emergent intubation cannot be ruled out. However, the analysis of GA by GA-policy vs. GA by CS-policy showed a higher proportion of good functional outcomes in GA by CS-policy, although it did not reach any statistical significance. Therefore, one can reasonably assume that the high rate of conversion had no or little impact on the results of our multivariate analysis, especially on the rates of favorable outcome at 90 days.

## Limitations

Our results should be viewed with caution. First, this study is a secondary *post-hoc* analysis, and the trial on which it is based was not designed to address the effects of GA-policy on clinical outcomes in AIS patients treated with MT. Indeed, the local anesthetic management policy was not randomized and did not include any active comparator group. Second, the used anesthetic agents and blood CO_2_ levels during procedure were not collected. Both factors are known to induce changes in cerebral autoregulation and therefore to impact on infarct core extension or functional outcome (24). Third, and above all else, the patient cohort in our study differs from those of previous published studies because of its high selection based on advanced imaging. Therefore, it formed a homogeneous group of good candidates for MT. This presented the advantage of limiting any sampling bias due to initial demographic heterogeneity and baseline stroke severity. However, it may also have minimized the eventual adverse impact of one of the anesthetic management policies in comparison with the other one. Fourth, our study was not powered to determine differences in adverse events or favorable clinical outcomes in subgroups e.g., based on anesthetic management. Therefore, these results need further larger studies.

## Conclusion

Our study suggests that both GA and CS when delivered as local routine management for patients undergoing MT present similar TTI and rates of successful revascularization, while GA delivered in CS-policy centers seems to be associated with longer ER arrival to reperfusion time. Nevertheless, GA might promoted lower rates of functional independence at 90 days based on the higher incidence of pneumonia and lower lowest procedural DPB values in the GA-policy group.

## Author contributions

OE, JS, MG, RJ, EL, RN, DY, and AB all participated in study design, data collection, and critical review and revision of the article. OE drafted the article.

### Conflict of interest statement

OE—UNRELATED: Consultancy: Stryker; Payment for Educational Presentations: Covidien; Payment for Development of Educational Presentations: Covidien, Medtronic, Stryker. AB—UNRELATED: Consultancy: Covidien; ev3; Grants/Grants Pending: Covidien (money paid to the institution). The remaining authors declare that the research was conducted in the absence of any commercial or financial relationships that could be construed as a potential conflict of interest.
