# Supplementary material for: Impact of Anesthetic Management on Safety and Outcomes Following Mechanical Thrombectomy for Ischemic Stroke in SWIFT PRIME Cohort
Source: Front Neurol. 2018 Aug 29;9:702. doi: 10.3389/fneur.2018.00702 (PMC6123376; doi:10.3389/fneur.2018.00702)
Supplement: Supplementary file 1 [file Table_1.docx]

Supplemental Table 1: Baseline, procedural, safety, and outcome data for the General Anesthesia patients and Conscious Sedation patients

| **Characteristics** | **Conscious Sedation patients (n=61)** | **General Anesthesia patients (n=36)** | **p-value** |
| --- | --- | --- | --- |
| *Baseline*  Age, yrs.  Male sex  NIHSS score  ASPECTS score  Infarct core volume, mL | 63.7 ± 12.6 (61)  59.0% (36/61)  16.6 ± 4.8 (61)  8.4 ± 1.5 (61)  8.5 ± 11.6 (57) | 67.4 ± 12.2 (36)  47.2% (17/36)  16.5 ± 4.2 (36)  8.4 ± 1.5 (36)  13.9 ± 20.2 (29) | 0.16  0.30  0.90  0.88  0.12 |
| *Risk factors*  Hypertension  Diabetes mellitus  Hyperlipidemia  Atrial fibrillation  Smoker | 57.4% (35/61)  16.4% (10/61)  24.6% (15/61)  31.1% (19/61)  41.0% (25/61) | 86.1% (31/36)  5.6% (2/36)  25.0% (9/36)  44.4% (16/36)  44.4% (16/36) | 0.004  0.20  1.00  0.20  0.83 |
| Glucose level, mg/dL | 127.6 ± 46.4 (61) | 136.9 ± 45.4 (35) | 0.34 |
| *Occlusion site*  ICA termination  MCA | 19.0% (11/58)  81.0% (47/58) | 18.2% (6/33)  81.8% (27/33) | 1.00  1.00 |
| *Procedural*  TTI, min  RT, min  LSBP, mmHg  LDBP, mmHg | 97.4 ± 42.2 (61)  42.6 ± 21.1 (52)  122.0 (77.0, 170.0) (55)  65.0 (40.0, 121.0) (55) | 102.8 ± 47.9 (36)  40.3 ± 14.9 (27)  110.0 (30.0, 150.0) (33)  55.0 (15.0, 77.0) (33) | 0.56  0.61  0.01  <0.001 |
| *Outcome*  TICI 2b/3  TICI 3  NIHSS score at 27 hrs  Infarct core volume at 27 hrs, mL  mRS 0–2 at 90 days  mRS 6 at 90 days | 78.7% (48/61)  62.3% (38/61)  7.7 ± 7.5 (61)  63.5 ± 98.1 (60)  62.3% (38/61)  8.2% (5/61) | 69.4% (25/36)  52.8% (19/36)  8.8 ± 6.7 (36)  49.5 ± 53.6 (36)  58.3% (21/36)  8.3% (3/36) | 0.34  0.40  0.45  0.43  0.83  1.00 |
| *Complications / Adverse events*  All ICH  Symptomatic ICH  SAH  PH2  Pneumonia  Vessel dissection | 26.2% (16/61)  0.0% (0/61)  1.6% (1/61)  6.6% (4/61)  13.1% (8/61)  1.6% (1/61) | 27.8% (10/36)  0.0% (0/36)  0.0% (0/36)  11.1% (4/36)  30.6% (11/36)  0.0% (0/36) | 1.00  1.00  1.00  0.46  0.06  1.00 |

ASPECT = Alberta Stroke Program Early Computed Tomography score; ICA = internal carotid artery; ICH = intracranial hemorrhage; LDBP = lowest diastolic blood pressure; LSBP = lowest systolic blood pressure; MCA = middle cerebral artery; mRS = modified Rankin score; NIHSS = National Institutes of Health Stroke Scale; PH2 = parenchymal hemorrhage grade 2 according to ECASS study ^17^; RT = reperfusion time; SAH = subarachnoid hemorrhage; sICH = symptomatic intracranial hemorrhage; TICI = thrombolysis in cerebral infarctions scale; TTI = time to treatment initiation; All values are shown as the mean ± SD (n), median (1^st^ and third interquartile) or percentage (n/N).
