# Supplementary material for: Impact of Anesthetic Management on Safety and Outcomes Following Mechanical Thrombectomy for Ischemic Stroke in SWIFT PRIME Cohort
Source: Front Neurol. 2018 Aug 29;9:702. doi: 10.3389/fneur.2018.00702 (PMC6123376; doi:10.3389/fneur.2018.00702)
Supplement: Supplementary file 2 [file Table_2.docx]

Supplemental Table 2: Perprocedural data in GA-policy and CS-policy groups after adjustment.

| **Procedural data** | **Mean difference** | **95%**  **Confidence Interval** | **p value** |
| --- | --- | --- | --- |
| TTI, min | -7.6 | -34.1, 18.8 | 0.57 |
| *Perprocedural*  LSBP, mmHg  LDBP, mmHg  Change from baseline SBP, %  Change from baseline DBP, % | -17.4  -14.5  -12.8  -14.2 | -29.9, -4.9  -22.3, -6.6  -23.0, -2.7  -25.2, -3.2 | 0.01  <0.001  0.02  0.01 |

DBP: diastolic blood pressure; LDBP = lowest diastolic blood pressure; LSBP = lowest systolic blood pressure; SBP: systolic blood pressure; TTI = time to treatment initiation.
