# Supplementary material for: Impact of Anesthetic Management on Safety and Outcomes Following Mechanical Thrombectomy for Ischemic Stroke in SWIFT PRIME Cohort
Source: Front Neurol. 2018 Aug 29;9:702. doi: 10.3389/fneur.2018.00702 (PMC6123376; doi:10.3389/fneur.2018.00702)
Supplement: Supplementary file 3 [file Table_3.docx]

Supplemental Table 3: Regression logistic model adjusted using the method of propensity scores comparing delivered GA with CS as the independent variable of interest

| **Outcome** | **Odds ratio*** | **95%**  **Confidence Interval** | **p-value** |
| --- | --- | --- | --- |
| *Efficacy*  TICI 2b/3  TICI 3  mRS 0–2 at 90 days | 0.87  0.81  0.31 | 0.18, 4.13  0.27, 2.48  0.10, 0.96 | 0.86  0.72  0.04 |
| *Safety*  SAH  PH  All ICH  mRS 6 at 90 days | 1.19  3.01  1.47  3.61 | NA  0.48, 18.62  0.47, 4.61  0.49, 26.77 | 1.00  0.24  0.51  0.21 |
| *Adverse events*  Pneumonia | 4.51 | 1.23, 16.58 | 0.02 |

ICH = intracranial hemorrhage; mRS = modified Rankin Scale; NA = not applicable; PH = parenchymal hematoma; SAH = subarachnoid hemorrhage; TICI = Thrombolysis in Cerebral Infarction scale. ^*^ The odds ratio reflects the odds of the General Anesthesia group in comparison with the Conscious Sedation group, so values < 1 indicate a reduced likelihood of the event with General Anesthesia compared with Conscious Sedation group.
